# Supplementary material for: Optimization of systemic AAV9 gene therapy in Niemann–Pick disease, type C1 mice
Source: Life Sci Alliance. 2026 Mar 30;9(6):e202402874. doi: 10.26508/lsa.202402874 (PMC13036363; doi:10.26508/lsa.202402874)
Supplement: Supplementary file 1 [file LSA-2024-02874_TableS1.docx]

**A**

| **Treatment** | **Sample Size (n)** | **Median Survival (weeks)** | **Significance**  **(Log-rank test)** |
| --- | --- | --- | --- |
| *Npc1^m1N^* Saline | 15 | 10.6 |  |
| *Npc1^m1N^* Low  7.87x10^12^ vg/kg | 10 | 11.4 | vs. Saline, ***P=0.0049*** |
| *Npc1^m1N^* Medium  1.28x10^14^ vg/kg | 24 | 21.5 | vs. Saline, ***P<0.0001***  vs. Low, ***P<0.0001*** |
| *Npc1^m1N^* High  3.06x10^14^ vg/kg | 8 | 34.6 | vs. Saline, ***P<0.0001***  vs. Low, ***P<0.0001***  vs. Medium, P=0.0266 |

**B**

| **Treatment** | **Sample Size (n)** | **Median Survival (weeks)** | **Significance**  **(Log-rank test)** |
| --- | --- | --- | --- |
| *Npc1^m1N^* Saline | 15 | 10.6 |  |
| *Npc1^m1N^* Med AAV9 at **4 weeks old** | 24 | 21.5 | vs. Saline, ***P<0.0001*** |
| *Npc1^m1N^* Med AAV9 at **6 weeks old** | 20 | 13.2 | vs. Saline, ***P<0.0001***  vs. 4 weeks old, ***P=0.0030*** |
| *Npc1^m1N^* Med AAV9 at **8 weeks old** | 20 | 11.9 | vs. Saline, ***P=0.0006***  vs. 4 weeks old, ***P<0.0001***  vs. 6 weeks old, ***P=0.0003*** |
