# Supplementary material for: Optimization of systemic AAV9 gene therapy in Niemann–Pick disease, type C1 mice
Source: Life Sci Alliance. 2026 Mar 30;9(6):e202402874. doi: 10.26508/lsa.202402874 (PMC13036363; doi:10.26508/lsa.202402874)
Supplement: Supplementary file 2 [file LSA-2024-02874_TableS2.docx]

**A**

| **Treatment** | **Sample Size (n)** | **Significance**  **(Tukey’s Multiple Comparisons Test, 6-9 weeks)** | **Significance**  **(Tukey’s Multiple Comparisons Test, 9-12 weeks)** |
| --- | --- | --- | --- |
| *Npc1^m1N^* Saline | 14 |  |  |
| *Npc1^m1N^* Low  7.87x10^12^ vg/kg | 10 | vs. Saline, P=0.9466 | vs. Saline, P=0.9976 |
| *Npc1^m1N^* Medium  1.28x10^14^ vg/kg | 13 | vs. Saline, P=0.0196  vs. Low, ***P=0.0069*** | vs. Saline, ***P<0.0001***  vs. Low, ***P<0.0001*** |
| *Npc1^m1N^* High  3.06x10^14^ vg/kg | 8 | vs. Saline, ***P<0.0001***  vs. Low, ***P<0.0001***  vs. Medium, P=0.0129 | vs. Saline, ***P<0.0001***  vs. Low, ***P<0.0001***  vs. Medium, ***P=0.0009*** |
| *Npc1^+/+^* | 21 | vs. Saline, ***P<0.0001***  vs. Low, ***P<0.0001***  vs. Medium, ***P=0.0003***  vs High, P=0.9104 | vs. Saline, ***P<0.0001***  vs. Low, ***P<0.0001***  vs. Medium, ***P<0.0001***  vs High, P=0.5073 |

**B**

| **Treatment** | **Sample Size (n)** | **Significance**  **(Tukey’s Multiple Comparisons Test, 6-9 weeks)** | **Significance**  **(Tukey’s Multiple Comparisons Test, 9-12 weeks)** |
| --- | --- | --- | --- |
| *Npc1^m1N^* Saline | 14 |  |  |
| *Npc1^m1N^* Med AAV9 at **4 weeks old** | 13 | vs. Saline, ***P<0.0001*** | vs. Saline, ***P<0.0001*** |
| *Npc1^m1N^* Med AAV9 at **6 weeks old** | 20 | vs. Saline, P=0.7788  vs. 4 weeks old, ***P=0.0010*** | vs. Saline, P=0.1909  vs. 4 weeks old, ***P=0.0002*** |
| *Npc1^m1N^* Med AAV9 at **8 weeks old** | 20 | vs. Saline, P=0.9984  vs. 4 weeks old, ***P<0.0001***  vs. 6 weeks old, P=0.5116 | vs. Saline, P>0.9999  vs. 4 weeks old, ***P<0.0001***  vs. 6 weeks old, P=0.0747 |
| *Npc1^+/+^* | 21 | vs. Saline, ***P<0.0001***  vs. 4 weeks old, P=0.2389  vs. 6 weeks old, ***P<0.0001***  vs 8 weeks old, ***P<0.0001*** | vs. Saline, ***P<0.0001***  vs. 4 weeks old, ***P<0.0001***  vs. 6 weeks old, ***P<0.0001***  vs 8 weeks old, ***P<0.0001*** |
