# Supplementary material for: Optimization of systemic AAV9 gene therapy in Niemann–Pick disease, type C1 mice
Source: Life Sci Alliance. 2026 Mar 30;9(6):e202402874. doi: 10.26508/lsa.202402874 (PMC13036363; doi:10.26508/lsa.202402874)
Supplement: Supplementary file 3 [file LSA-2024-02874_TableS3.docx]

**A**

| AAV9 Dose Study (Sample size for weight curves) | | | | | |
| --- | --- | --- | --- | --- | --- |
| Treatment | *Npc1^m1N^* saline | *Npc1^m1N^* low | *Npc1^m1N^* med | *Npc1^m1N^* high | *Npc1^+/+^* untreated |
| Male | 8 | 6 | 10 | 4 | 11 |
| Female | 7 | 4 | 14 | 4 | 10 |
| **Total** | **15** | **10** | **24** | **8** | **21** |

**B**

| AAV9 Age at Injection Study (Sample size for weight curves) | | | | | |
| --- | --- | --- | --- | --- | --- |
| Treatment | *Npc1^m1N^* saline | *Npc1^m1N^* med 4 wks | *Npc1^m1N^* med 6 wks | *Npc1^m1N^* med 8 wks | *Npc1^+/+^* untreated |
| Male | 8 | 10 | 11 | 12 | 12 |
| Female | 7 | 14 | 9 | 8 | 10 |
| **Total** | **15** | **24** | **20** | **20** | **22** |

**C**

| AAV9 Hypomorphic (I1061T) Model Study (Sample size for weight curves) | | | |
| --- | --- | --- | --- |
| Treatment | *Npc1^I1061T^* saline | *Npc1^I1061T^* med | *Npc1^+/+^* untreated |
| Male | 5 | 7 | 6 |
| Female | 6 | 8 | 9 |
| **Total** | **11** | **15** | **15** |
