# Supplementary material for: Optimization of systemic AAV9 gene therapy in Niemann–Pick disease, type C1 mice
Source: Life Sci Alliance. 2026 Mar 30;9(6):e202402874. doi: 10.26508/lsa.202402874 (PMC13036363; doi:10.26508/lsa.202402874)
Supplement: Supplementary file 4 [file LSA-2024-02874_TableS4.docx]

| Antibody | Company | Catalog Number | Dilution | Method |
| --- | --- | --- | --- | --- |
| α-β-actin (mouse IgG) | ThermoFisher Invitrogen | 15G5A11/E2 | 1:10000 | western blot |
| α-Calbindin (mouse IgG) | Sigma | C9848 | 1:750 1:1000 | IF western blot |
| α-Calbindin (rabbit IgG) | Abcam | ab229915 | 1:750 (fixed, free-floating or FFPE sections) | IF |
| α-CD68 (rat IgG2a) | BioRad | MCA1957 | 1:500 | IF |
| α-CD68 (rabbit IgG) | Abcam | ab125212 | 1:1000 | IHC, IF, western blot |
| α-GFAP (mouse IgG) | Sigma | G3893 | 1:1000 (fixed, free-floating or FFPE sections)  1:2000 | IF  western blot |
| α-IBA1 (rabbit IgG) | Wako Chemicals | 019-19741 | 1:500 (fixed, free-floating sections) or 1:750 (FFPE) | IF |
| α-NPC1 (monoclonal rabbit IgG) | Abcam | ab134113 | 1:2000 | western blot |
| α-β-III-Tubulin (monoclonal mouse IgG) | R&D Systems | MAB1195 | 1:3000 | western blot |
| Donkey anti-mouse or anti-rabbit IgG IRDye 680RD | LI-CORbio | 926-68072 or 926-68073 | 1:20000 | western blot |
| Donkey anti-mouse or anti-rabbit IgG IRDye 800CW | LI-CORbio | 926-32212 or 926-32213 | 1:20000 | western blot |
| Goat anti-rabbit IgG Biotinylated | Vector Laboratories | BA-1000 | 1:300 | IHC |
| Goat anti-mouse IgG AlexaFluor 488 or 594 | ThermoFisher Invitrogen | A11029 (488) or A11005 (594) | 1:350 | IF |
| Goat anti-rabbit IgG AlexaFluor 488 or 594 | ThermoFisher Invitrogen | A11034 (488) or A11037 (594) | 1:350 | IF |
| Goat anti-rat IgG Alexa Fluor 488 or 594 | ThermoFisher Invitrogen | A11006 (488) or A11007 (594) | 1:350 | IF |
